# The connection between professional burnout of medical workers and the specific working conditions during the COVID-19 pandemic in Russia

**DOI:** 10.1192/j.eurpsy.2024.1058

**Published:** 2024-08-27

**Authors:** E. V. Deshchenko, J. E. Koniukhovskaia, O. B. Stepanova, I. M. Shishkova, E. I. Pervichko, O. V. Mitina, E. A. Dorokhov

**Affiliations:** ^1^ Lomonosov Moscow State University; ^2^Higher School of Economics, Moscow; ^3^Ryazan State Medical University, Ryazan, Russian Federation

## Abstract

**Introduction:**

The COVID-19 pandemic has certainly become a stressful event for medical workers, so the aim of this research was to study the pandemic-specific working conditions that may be associated with the professional burnout of medical workers in Russia.

**Objectives:**

To study the pandemic-specific working conditions that may be associated with the professional burnout of medical workers in Russia.

**Methods:**

The Maslach Burnout Inventory (MBI) was used to measure the level of professional burnout. It was filled out by medical workers from January 2021 to November 2022.

The sample consisted of 314 medical workers (57 men and 255 women), whose average age was 36.97±11.93. According to the level of education, the sample included specialists with secondary general education (4.14%), with secondary special education (19.4%), with incomplete higher education (11.46%), with higher education (59.87%) and PhD (5.1%). 35 people (11%) of the surveyed medical workers worked in the red zone.

**Results:**

Working in the red zone is significantly associated with Emotional Exhaustion (p=0.002) and Depersonalization (p=0.002), but not with a Reduction in Professionalism.

The working conditions of medical workers who were significantly associated simultaneously with Emotional Exhaustion, Depersonalization and Reduction of professionalism (respectively): (1) Lack of confidence in support from the health system and the state in case of illness (r=0.170, p=0.002; r=0.202, p=0.000; r=-0.171, 0. 002); (2) Inability to meet the usual personal needs (daily routine, nutrition, communication with loved ones) as employment increases at work (r=0.200, p=0.000; r=0.154, p=0.006; r=-0.186, 0. 001); (3) Lack of confidence in their own professional competence in the fight against COVID-19 due to lack of knowledge about COVID-19 (r=0.202, p=0.000; r=0.148, p=0.009; r=-0.211, 0. 000); (4) Lack of confidence in their own effectiveness in the fight against COVID-19 due to the increase in the volume of work and the expansion of the scope of professional responsibilities (r=0.234, p=0.000; r=0.152, p=0.007; r=-0.177, 0. 002); (5) Lack of access to up-to-date information about COVID-19 (r=0.190, p=0.001; r=0.158, p=0.005; r=-0.140, 0. 013).

The Emotional Exhaustion scale is also associated with the fear of getting infected and getting sick with COVID-19 (r=0.125; p=0.026), as well as the lack of quick access to testing when COVID-19 symptoms appear (r=0.169; p=0.003).

**Conclusions:**

Thus, not only work in the red zone, but also many specific working conditions during the COVID-19 pandemic can become a provocateur factor for the deterioration of the emotional state of medical workers.

Disclosure: Research is supported by the Russian Science Foundation, project No. 21-18-00624.

**Disclosure of Interest:**

None Declared

